# Residual Flexural Behavior of Hybrid Fiber-Reinforced Geopolymer After High Temperature Exposure

**DOI:** 10.3390/ma18153572

**Published:** 2025-07-30

**Authors:** Yiyang Xiong, Ruiwen Jiang, Yi Li, Peipeng Li

**Affiliations:** School of Civil Engineering and Architecture, Wuhan University of Technology, Wuhan 430070, China

**Keywords:** geopolymer, flexural behavior, hybrid fiber, high temperature resistance

## Abstract

Cement-based building materials usually exhibit weak flexural behavior under high temperature or fire conditions. This paper develops a novel geopolymer with enhanced residual flexural strength, incorporating fly ash/metakaolin precursors and corundum aggregates based on our previous study, and further improves flexural performance using hybrid fibers. The flexural load–deflection response, strength, deformation capacity, toughness and microstructure are investigated by a thermal exposure test, bending test and microstructure observation. The results indicate that the plain geopolymer exhibits a continuously increasing flexural strength from 10 MPa at 20 °C to 25.9 MPa after 1000 °C exposure, attributed to thermally induced further geopolymerization and ceramic-like crystalline phase formation. Incorporating 5% wollastonite fibers results in slightly increased initial and residual flexural strength but comparable peak deflection, toughness and brittle failure. The binary 5% wollastonite and 1% basalt fibers in geopolymer obviously improve residual flexural strength exposed to 400–800 °C. The steel fibers show remarkable reinforcement on flexural behavior at 20–800 °C exposure; however, excessive steel fiber content such as 2% weakens flexural properties after 1000 °C exposure due to severe oxidation deterioration and thermal incompatibility. The wollastonite/basalt/steel fibers exhibit a positive synergistic effect on flexural strength and toughness of geopolymers at 20–600 °C.

## 1. Introduction

Cement-based materials are widely used in construction and building engineering due to the abundance of raw materials, relatively low cost and excellent performance and service life. However, the normal cementitious mortar and concrete usually suffer from severe deterioration and damage when exposed to high temperatures or extreme fire conditions [1,2,3,4,5,6]. This damage is primarily caused by the decomposition of the hydration products, thermal incompatibility of different phases, high pore vapor pressure and phase transition of aggregates [2]. Consequently, the bearing capacity, crack resistance and fire resistance limit of the components and structure would be greatly weakened [7]. Furthermore, the use of Portland cement results in high CO_2_ emission and energy consumption, leading to environmental issues in the construction sector. Thus, it is necessary to develop a novel high temperature-resistant and environmentally friendly building material.

Geopolymer was conceptually proposed by Joseph Davidovits in 1978, with cementless binders as precursors such as fly ash, slag and metakaolin [8]. It is typically activated using an alkaline or acidic solution and generates a three-dimensional network structure. Compared with normal cement-based materials, geopolymer offers much greater sustainability by utilizing industrial by-products and significantly reducing carbon emissions [9,10,11]. The performance of this new material is characterized by high early-age strength, superior chemical resistance and durability, as well as excellent high temperature resistance [9,12]. However, current studies usually focus on a single precursor geopolymer that sometimes has disadvantages; for example, fly ash-based geopolymer can maintain a high residual strength retention rate but achieves a relatively slow hardening process and low initial strength. Furthermore, silica sands and limestone aggregates are frequently utilized in geopolymer concrete, which certainly cause phase transition and decomposition under high temperatures [13]. Additionally, much of the literature demonstrates the thermal stress and incompatibility between normal aggregates and matrices [1]. Therefore, optimized blended precursors and thermally stable aggregates have potentially positive effects on developing high temperature-resistant geopolymer.

Plain geopolymer has a comparable brittle performance as a cement-based material, and a relatively weak flexural behavior compared with its compressive properties. The fiber utilization could remarkably address the brittle behavior and reinforce the flexural properties of both normal concrete and geopolymer [9,14,15,16]. Introducing fibers in concrete inhibits crack propagation and strengthens the flexural, tensile, energy absorption, ductility and toughness [17]. Li et al. improved the static and dynamic flexural behavior of high performance concrete with steel fiber [15,16]. A multiscale investigation indicated increments in the pullout load and energy of fiber/matrix bonding and, thus, the residual flexural strength and energy absorption of fiber reinforced the cementitious composites after 200–400 exposures [18]. Zhao et al. pointed out that the geopolymer matrix designs, fiber types and content could significantly affect the fire response of fiber-reinforced geopolymer [12]. The brittle failure of ordinary Portland cement concrete and geopolymer concrete was successfully transferred to ductile damage at ambient and high temperatures [14]. However, different fiber types probably lead to quite different reinforcements on the residual flexural behavior of geopolymer. Polyvinyl alcohol (PVA) fiber might be melted at relatively high temperatures and exhibits limited enhancement compared with polyethylene (PE) fiber [19,20], while the residual strength of carbon fiber-reinforced geopolymer first increases and then decreases, achieving an optimal fiber content of 0.6% [21]. Vijaya Prasad et al. [17] compared crimped steel, PE and basalt fibers in high temperature-resistant geopolymer, and indicated that the optimized hybrid fiber combination could further improve the residual strength and interfacial bond. The interfacial bond and pullout performance between the fiber and matrix can be enlarged under a higher loading rate [22], and the flexural strength and energy absorption of concrete is increased with the increase in the flexural loading rate [23,24]. However, there has been limited investigation into the ternary hybrid fibers in high temperature- or fire-resistant geopolymer, especially for fibers with different types and scales, such as steel, basalt and wollastonite fibers.

Our previous research successfully developed a novel ultra-high residual compressive strength enhanced geopolymer after high temperature exposure [25,26]. The blended fly ash/metakaolin binder contributed to both high initial and residual compressive strength, and corundum aggregates could avoid the matrix thermal crack and result in very high residual compressive strength. However, its flexural behavior has not been investigated, especially under fiber reinforcement. This study aims to develop a geopolymer with enhanced residual flexural strength after high temperature exposures, using fly ash/metakaolin blended precursors and corundum aggregates. Furthermore, the use of wollastonite/basalt/steel fibers is proposed to improve flexural performance. Flexural, thermal exposure and SEM tests are conducted to evaluate load–deflection response, flexural strength, deformation capacity, toughness and microstructure characteristics.

## 2. Materials and Methods

### 2.1. Raw Materials

**Figure 1:**
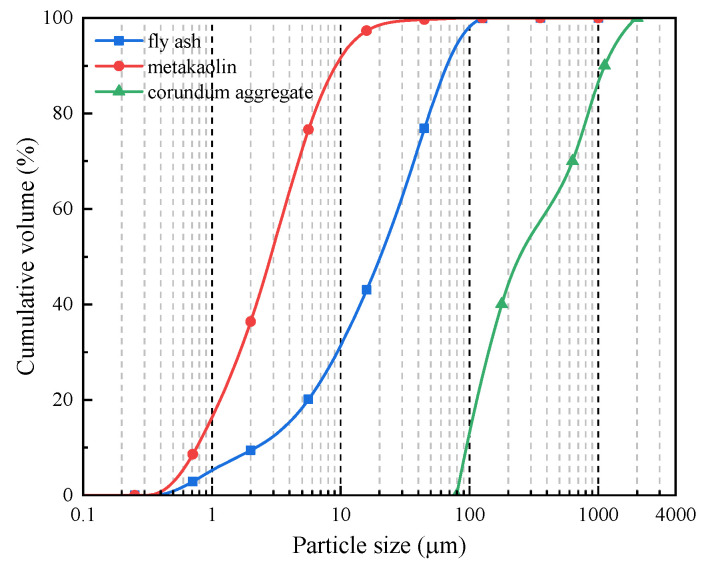
Particle size distribution of binders and aggregate.

**Figure 2:**
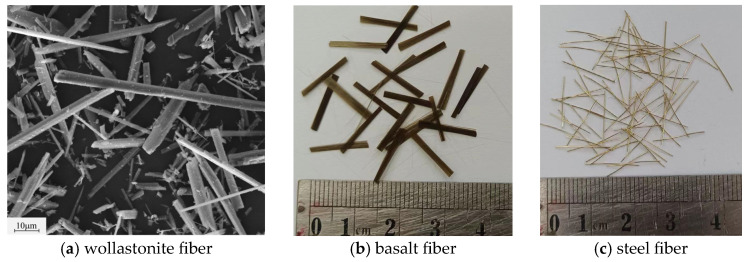
Images of (**a**) wollastonite, (**b**) basalt and (**c**) steel fibers.

The utilized binders include F-class fly ash (FA) and metakaolin (MK) as precursors. The alkali activator is composed of potassium hydroxide (99% KOH) and potassium silicate (water glass, 26.88% SiO_2_ and 12.75% K_2_O). Corundum aggregates (CDAs) with Al_2_O_3_ content more than 99% are employed due to their inherent high temperature resistance and thermal compatibility with geopolymer matrix [25]. To enhance the flexural behavior at both micro and macro levels, wollastonite fibers (WFs), steel fibers (SFs) and basalts fibers (BFs) are incorporated into the geopolymer. Wollastonite is a naturally fibrous calcium silicate mineral with a needle-like crystal structure, and its chemical composition is *β*-CaO-SiO_2_. The basalt fibers used have a length of 12 mm and diameter of 17 μm. The steel fibers are short straight fibers with a length of 13 mm and diameter of 0.2 mm. The chemical compositions of binders and wollastonite are presented in Table 1 [25,26,27]. The particle size distribution of binders and aggregates are shown in Figure 1, and the images of different fibers are provided in Figure 2.

### 2.2. Mix Design and Preparation

Table 2 presents the seven mix designs of high temperature-resistant geopolymers. The reference mixture, based on our previous studies, demonstrates excellent residual compressive strength after high temperature exposure. It consists of optimized fly ash/metakaolin blended precursors, a potassium silicate alkali activator and corundum aggregates [25,27]. The fly ash (FA) to metakaolin (MK) ratio is 7:3. The alkali activator is potassium silicate solution (water glass, WG), containing 49.09% wt% K_2_O∙SiO_2_ with a modulus of 1.0. Additionally, 99.9 kg/m^3^ of tap water is added to achieve the desired workability. The key research parameters include the wollastonite fiber, basalt fiber, steel fiber and their hybrid utilization. The wollastonite fiber content is calculated based on the mass of total precursors, while the basalt and steel fiber contents are measured by the volume percentages of total geopolymer mixture. The mix codes represent the fiber type and volume content percentage, e.g., F0 refers to the reference geopolymer without fiber, while W5B0.5S1 represents the fiber-reinforced geopolymer mix with 5% wollastonite fiber, 0.5% basalt fiber and 1% steel fiber.

The fly ash and metakaolin are first dry mixed, followed by adding water glass and tap water, then the corundum aggregates. The fresh geopolymer mortars are cast into 40 mm × 40 mm × 160 mm prismatic molds and covered with plastic film. After 24 h, the hardened specimens are demolded and cured for 28 days at a temperature of 20 ± 2 °C and relative humidity ≥ 95%, in preparation for further high temperature exposure, flexural and SEM testing.

### 2.3. Testing Methods

A MF-1200C muffle furnace is used to conduct the high temperature exposure test. The geopolymer specimens cured for 28 days are placed in the furnace, with a heating rate of 10 °C/min. The target temperatures of 200 °C, 400 °C, 600 °C, 800 °C and 1000 °C are set and maintained for 1 h. After heating, the furnace is turned off and the specimens are allowed to naturally cool down to room temperature.

The three-point bending test is conducted on the 40 mm × 40 mm × 160 mm prismatic specimens, both at room temperature and after high temperature exposure. The flexural test is conducted with a 300 kN mortar testing machine and controlled by displacement loading at a 50 N/s loading rate, and stopped upon complete failure or when the displacement reaches approximately 3.5 mm. The load and displacement data are recorded, and the corresponding key flexural properties are calculated and derived.

The small piece geopolymer samples, both before and after high temperature exposures, are immersed in absolute ethanol for 24 h, dried at 60 °C for 24 h and then coated with Au. The scanning electron microscopy (SEM) test by TESCAN MIRA LMS is carried out to observe the morphologies and microstructures of geopolymerization products and interfacial interactions between the fibers and the matrix.

## 3. Results and Discussions

### 3.1. Damage and Flexural Load–Deflection Response

**Figure 3:**
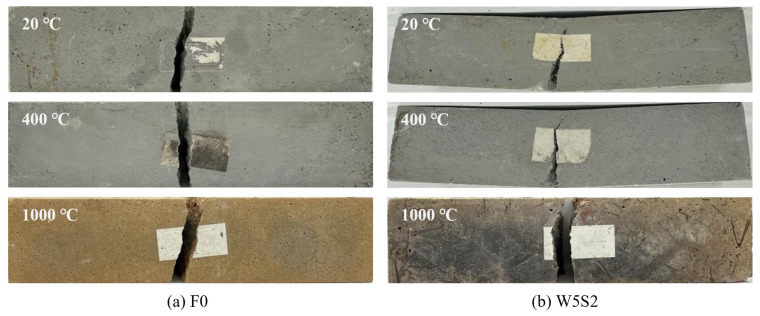
Typical damage patterns of geopolymers under after different temperature exposures: (**a**) F0 and (**b**) W5S2.

Figure 3 presents the two typical damage patterns and surface morphology changes under characteristic temperatures, including brittle and ductile flexural breakages. Notably, all the designed geopolymers exhibit excellent crack resistance under high temperature exposures even up to 1000 °C. No obvious visible surface crack or any high temperature-induced explosive spalling can be detected. By comparison, the cement-based concrete usually suffers from severe crack damage after high temperature exposure above 800 °C [13]. Because of the internal moisture evaporation, the surface color of the geopolymer matrix varies from dark gray at 20 °C to light gray at 400 °C, and it further gradually changes to dark brown with increasing temperature up to 1000 °C due to viscous sintering [25,28,29]. The surface color of geopolymer reinforced with steel fiber, e.g., W5S2, tends to be black, attributed to steel fiber oxidation. The plain, wollastonite and basalt fiber-reinforced geopolymer show a brittle flexural rupture, as illustrated in Figure 3a. While, as shown in Figure 3b, for geopolymer W5S2, the steel fiber incorporation provides for an excellent bridge effect and usually results in a ductile flexural damage pattern, but it becomes brittle again after the steel fiber complete oxidation at 1000 °C.

**Figure 4:**
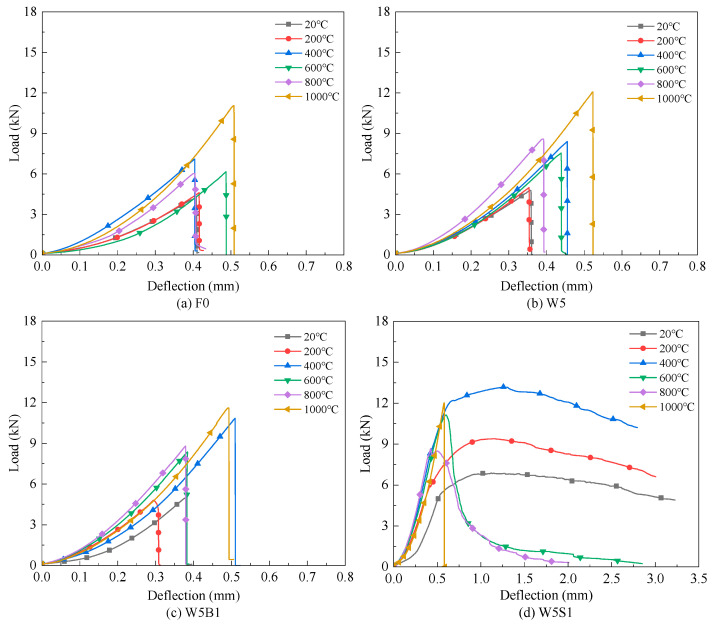

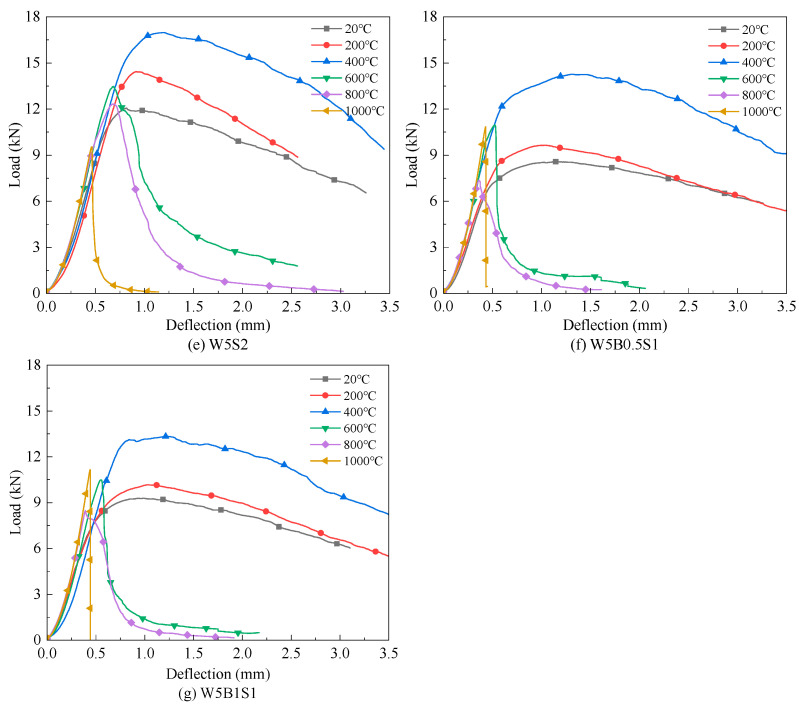
Flexural load–deflection curves: (**a**) F0, (**b**) W5, (**c**) W5B1, (**d**) W5S1, (**e**) W5S2, (**f**) W5B0.5S1 and (**g**) W5B1S1.

Figure 4 shows the flexural load–deflection curves of the designed reinforced geopolymers with different fiber reinforcements. The reference plain geopolymer presents typical brittle flexural behavior both before and after high temperature exposure, characterized by an initial elastic rise followed by a sudden drop, as shown in Figure 4a. It is worth noting that the mix design of the plain geopolymer was optimized using metakaolin/fly ash blended precursors, potassium silicate alkali activators and thermally resistant corundum aggregates based on our previous study [25]. Interestingly, the plain geopolymer demonstrates a continuously enhanced flexural load–deflection response with increasing exposure temperature. This behavior mirrors the development observed in its residual compressive strength [25]. The compressive strength of the 40 mm × 40 mm × 40 mm cubic plain geopolymer specimen after the same high temperature exposure as this study is enhanced from 50.9 MPa at 20 °C to 106.8 MPa at 1000 °C. In contrast, the conventional cement-based building materials usually experience a severe residual flexural behavior reduction because of raw ingredient thermal incompatibility, hydration product decomposition and extreme pore pressure, especially above the 600 °C temperature exposures [1,13,14]. The increased flexural behavior after 200 °C and 400 °C is attributed to the further geopolymerization reactions of the unreacted precursors [27,30], as can be seen in the SEM observation below. However, after exposure to 600 °C and 800 °C, the flexural load–deflection curve maintains a similar response or even a slight decrease due to the comprehensive effects of positive further geopolymerization and negative high temperature deterioration, including gel dehydration, dihydroxylation reaction-bound water loss, thermal expansion incompatibility, etc. After being exposed to 1000 °C, the viscous sintering occurs and generates ceramic-like crystalline phases [25,26], which result in the significant enhancement of flexural load–deflection response.

As a microscale mineral fiber, wollastonite exhibits an excellent inherent thermal stability [31]. When 5% wollastonite fiber is used, the flexural load–deflection curves at both ambient temperature and after high temperatures are very similar but slightly enhanced, as shown in Figure 4b. With the addition of basalt fiber alongside wollastonite (binary reinforcement), the brittle flexural response is still observed, but the residual flexural response could be enlarged, especially after 400 °C exposure, as illustrated in Figure 4c. The basalt fibers are characterized by a relatively low elastic modulus and high melting temperature, which could retain some bridge effect and crack resistance at elevated temperature [7,17]. In addition, Figure 4d,e present the synergistical utilization wollastonite and steel fibers, and the geopolymer exhibits significant improvement in the flexural load–deflection response both before and after high temperature exposures. Notably, the positive reinforced effect is more profound at ambient and relatively lower temperatures between 20 °C and 400 °C. As a fiber with high tensile strength and elastic modulus, the steel fiber contributes positively to both compressive and flexural strength [32,33]. However, its performance deteriorates at higher temperatures due to oxidation, resulting in reduced effectiveness or even a slightly negative impact between 600 °C and 1000 °C. Compared with mixture W5S1, which contains 1% steel fiber, mixture W5S2 with 2% steel fiber shows an even greater reinforcement effect. Nevertheless, using excessive volumes of steel fiber is not recommended, as it complicates mixing and casting, increases cost and leads to a diminished performance at very high temperatures (e.g., 1000 °C).

The above analysis of single and binary fiber reinforcement effects indicates that different types and scales of fiber could improve flexural behavior across different temperature ranges and cracking mechanisms. Thus, incorporating a ternary hybrid fiber system with wollastonite, basalt and steel systems could probably reinforce the flexural behavior across a full range of temperatures. Figure 4f shows enhancement of the flexural behavior of W5B0.5S1 at ambient temperature and 400 °C but comparable flexural responses at other temperatures compared with mixture W5B1. Furthermore, more additional basalt fiber in W5B1S1 cannot further promote flexural behavior, as seen in Figure 4g, probably attributed to fiber agglomeration and flowability reduction in the case of a too high total fiber content.

### 3.2. Flexural Strength

**Figure 5:**
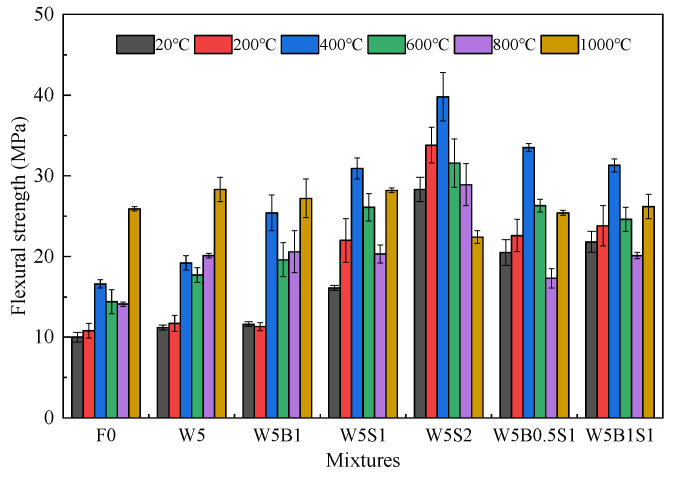
Flexural strength of geopolymer before and after high temperature exposure.

Flexural strength, an important indicator of bending performance, is derived from the peak load and represents the maximum load-bearing capacity. Figure 5 shows the flexural strength of all geopolymer specimens before and after high temperature exposure. The initial flexural strength at ambient temperature is around 10 MPa, then continuously gradually enhances to approximately 16.6 MPa at 400 °C exposure. However, under higher exposure temperatures such as 600 °C and 800 °C, the flexural strength adversely experiences a slight reduction. Notably, a significantly enlarged residual flexural strength of 25.9 MPa can be obtained after exposure to 1000 °C. It is approximately 2.6 times the initial ambient flexural strength, which is attributed to the viscous sintering and ceramic-like crystalline phase formation, as well as the relatively excellent thermal compatibility between the corundum aggregates and the matrix [25,26]. By incorporating 5% wollastonite fibers in geopolymer, the ambient temperature flexural strength is increased to 11.2 MPa, and the residual flexural strength experiences obvious improvements after exposure to 400–1000 °C. With the further addition of 1% basalt fibers in the W5B1 mix, most flexural strengths are comparable to W5, while the residual flexural strength could be greatly improved after moderate temperature exposures, namely 25.4 MPa at 400 °C and 19.6 MPa at 600 °C. The flexural strength of W5S1 varies between 16.1 MPa, 22.0 MPa, 30.9 MPa, 26.1 MPa, 20.3 MPa and 28.2 MPa, based on the temperature between 20 °C and 1000 °C. Additionally, the flexural strength of W5S2 with more steel fibers exhibits a continuous increase from 28.3 MPa at 20 °C to 39.8 MPa at 400 °C, then continuously reduces to 22.4 MPa. It indicates that steel fibers can efficiently reinforce flexural strength between 20 °C and 600 °C, or even 800 °C. But steel fiber utilization does not show any positive effect on residual flexural strength after 1000 °C exposure, even having a negative effect in the case of a too high volume content such as 2% because of severe fiber deterioration [1,32]. Compared with the binary fiber reinforced in W5B1 and W5S1, utilizing ternary hybrid fibers in W5B0.5S1 and W5B1S1 shows a comprehensively good performance, namely greatly enhanced flexural strengths during 20–600 °C and comparable properties during 800–1000 °C.

As comparison, the normal cement-based concretes usually possess relatively weak residual flexural strength after high temperature exposure [1,14,34,35,36]. The residual flexural strength beyond 600 °C temperature of normal plain concrete usually remains less than 50% of the initial strength at ambient temperature. For example, the flexural strength changes from 5.8 MPa at 20 °C to only 1.5 MPa at 600 °C in reference [1], while it can be improved to 15 MPa and 9.2 MPa with 2% steel fiber, respectively. Another research indicated that the flexural strength experienced a continuous decrease from 5.3 MPa to 1.0 MPa up to 800 °C exposure, and the utilization of hybrid PVA and steel fibers enhanced the flexural strength at ambient and 800 °C temperature to 7.2 MPa and 1.6 MPa, respectively.

**Figure 6:**
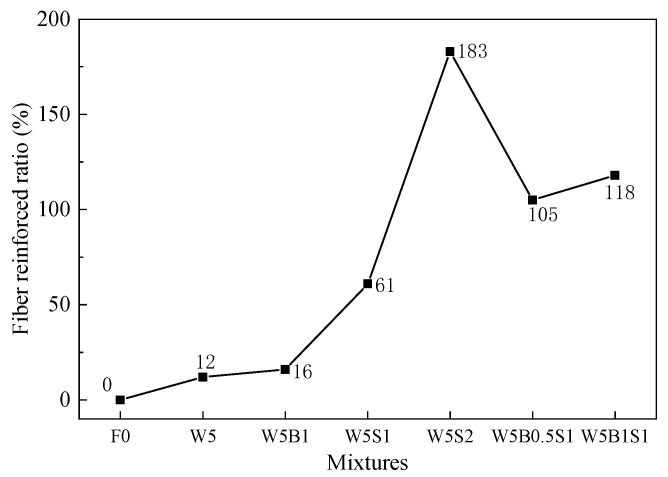
Fiber-reinforced ratio of flexural strength of geopolymer at ambient temperature.

Figure 6 shows the fiber-reinforced ratio of the flexural strength of geopolymer composites at ambient temperature, representing the absolute increasement percentage compared with the plain mix. All fiber-reinforced cases exhibit clear improvements in flexural strength, primarily due to the crack-bridging effect and pullout behavior provided by fibers at the early stages of crack development [33]. The incorporation of 5% wollastonite fibers results in a reinforced ratio of 12%, and the binary fiber utilization obtains further enhancement, with reinforced ratios of 16%, 61% and 183% for the mixtures of W5B1, W5S1 and W5B2, respectively. In the presence of ternary hybrid fibers, the increasement on flexural strength reaches 105% and 118%. Notably, the fiber-reinforced ratio of 118% for W5B1S1 is much higher than the sum-reinforced ratio of W5B1 and W5S1, which demonstrates an apparently positive synergistic effect of the ternary hybrid fiber addition on the flexural strength at ambient temperature.

**Figure 7:**
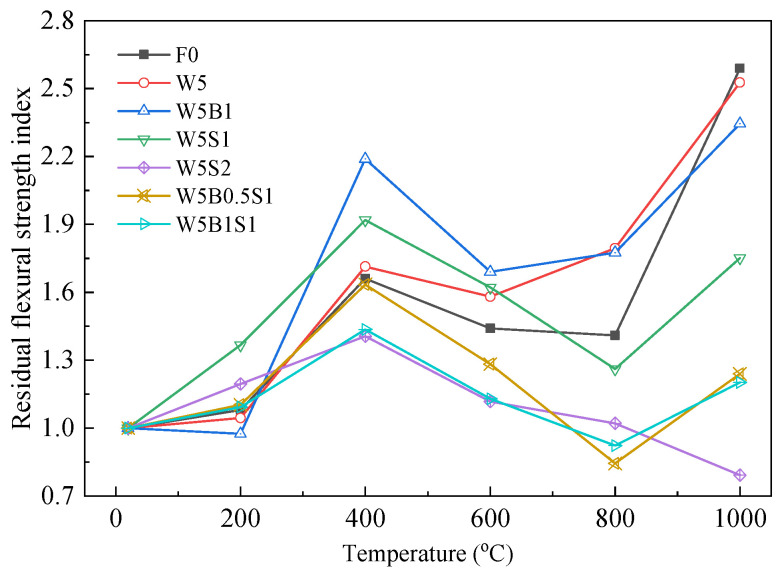
Residual flexural strength index of geopolymer after high temperature exposure.

The residual flexural strength index is defined as the ratio of the flexural strength after high temperature exposure to the initial flexural strength at ambient temperature. A higher residual flexural strength index represents a better residual bearing capacity and a greater safety margin under extreme thermal conditions. Figure 7 illustrates the residual flexural strength index of geopolymer after high temperature exposure. All geopolymer specimens possess increased residual flexural strength indices below 400 °C due to the further geopolymerization reaction of the unreacted precursors under high temperature conditions, especially for the mixture W5B1, with an index at approximately 2.2. Then, the indices usually decrease until 800 °C because of both matrix and interfacial transition zone (ITZ) deteriorations, as well as probable fiber degradation. Subsequently, the residual flexural strength indices of most geopolymers, except for W5S2, sharply increase again when exposed to 1000 °C, particularly geopolymers F0, W5 and W5B1 without any steel fiber. However, introducing an excessive volume content such as 2% steel fiber tends to negatively affect the residual flexural strength indices. In summary, most of the fiber-reinforced geopolymers developed in this study exhibit both high residual flexural strength and favorable residual flexural strength index values. These characteristics indicate a strong potential for application in fire-resistant and high temperature structural systems, even without requiring post-fire repair.

### 3.3. Deformation Capacity

**Figure 8:**
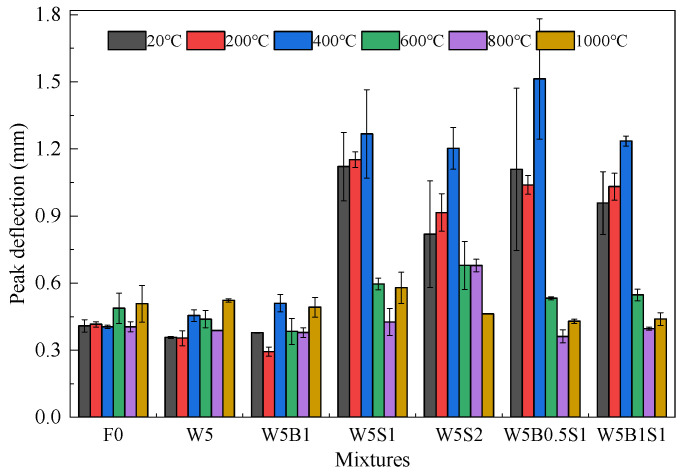
Peak deflection of geopolymer before and after high temperature exposure.

The peak deflection, defined as the displacement corresponding to the peak load, is used to analyze the deformation capacity of geopolymers. Figure 8 exhibits the peak deflection of various geopolymer mixtures before and after high temperature exposure. The plain geopolymer shows relatively small peak deflections, indicating limited deformation capacity. The incorporation of wollastonite and basalt fibers does not significantly improve the deformation capacity. Across all temperature exposures, the peak deflections of F0, W5 and W5B1 generally fluctuate approximately between 0.3 mm and 0.5 mm, which is consistent with their brittle flexural failures. In contrast, the addition of steel fibers contributes to greatly enlarged peak deflections, especially during the 20–400 °C exposures, with several times higher peak deflections than these of the brittle mixtures. For instance, the peak deflection of W5S1 increases from 1.12 mm at 20 °C to 1.27 mm at 400 °C and finally 0.58 mm at 1000 °C, while W5B0.5S1 achieves even further notable deformation ability after 400 °C exposure, namely around 1.51 mm for peak deflection.

### 3.4. Flexural Toughness

**Figure 9:**
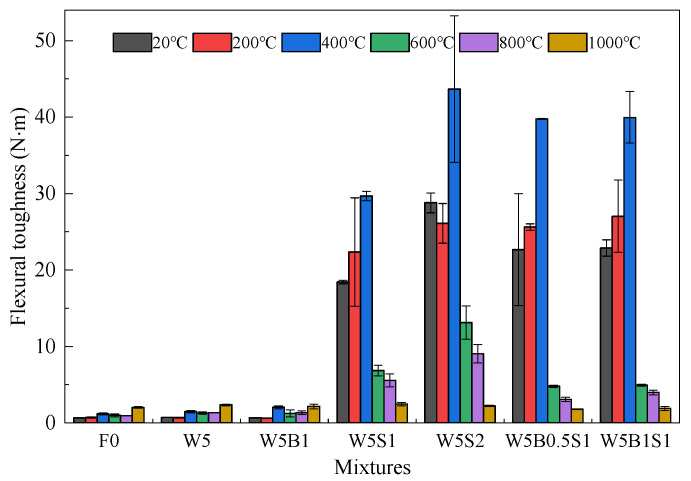
Flexural toughness of geopolymer before and after high temperature exposure.

The flexural toughness in Figure 9 is calculated by the envelope area on the flexural load–displacement curve, which represents energy absorption capacity and ductility [18]. Due to its brittle flexural failure, the reference mixture F0 exhibits very weak flexural toughness, ranging from 0.69 N∙m at 20 °C to 2.03 N∙m at 1000 °C. Incorporating wollastonite and basalt fibers, as in mixes W5 and W5B1, results in only minor improvements in the flexural toughness compared with that of F0. However, the addition of 1% steel fibers and 5% wollastonite fibers in W5S1 greatly contributes to enlarged flexural toughness, particularly 18.4–29.7 N∙m during 20–400 °C. The enhanced flexural toughness even reaches 28.8–43.7 N∙m in the presence of 2% steel fibers. The pullout process of macro basal or steel fibers from the matrix could greatly improve energy absorption and then enlarge flexural toughness [22,24]. In the case of ternary hybrid fibers in W5B0.5S1 and W5B1S1, flexural toughness below 400 °C exposure is apparently higher than the corresponding toughness of W5S1. It demonstrates a positive synergistic effect of the ternary hybrid fibers on the flexural toughness of the designed geopolymer, which is similar to the trend in flexural strength.

### 3.5. SEM Analysis

**Figure 10:**
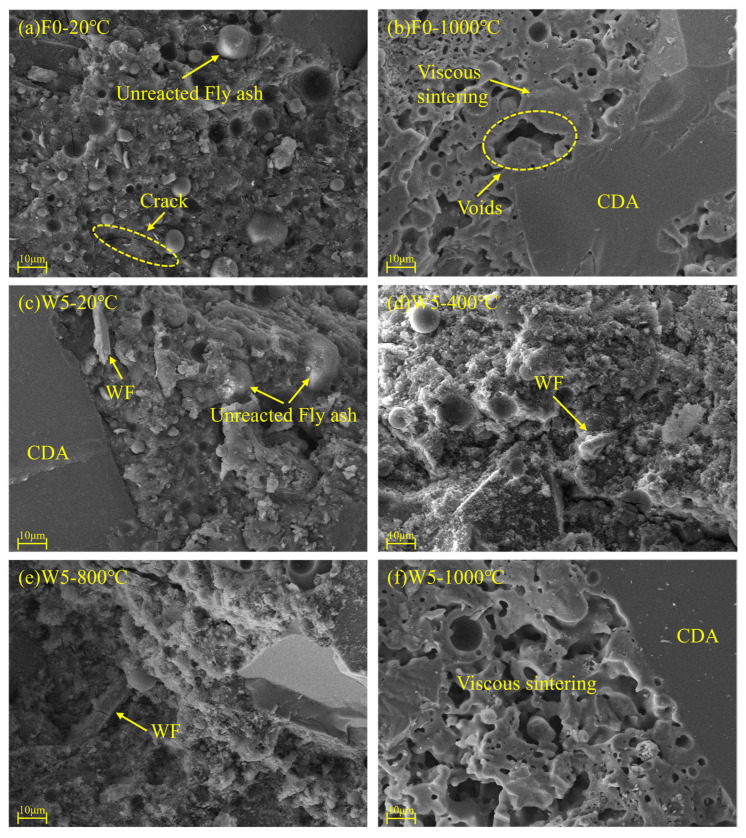
SEM morphologies of geopolymer without fiber and with wollastonite fibers: (**a**) F0-20°C, (**b**) F0-1000°C, (**c**) W5-20°C, (**d**) W5-400°C, (**e**) W5-800°C and (**f**) W5-1000°C.

The microstructure and reaction products of geopolymer are closely linked to flexural behavior. Figure 10 shows the SEM morphologies of geopolymer without fiber and with wollastonite fibers. After 28 days of ambient curing, the geopolymer has a relatively dense microstructure but still retains some unreacted precursor particles such as fly ash, as illustrated in Figure 10a,c. These unreacted particles result in further geopolymerization after high temperature exposure, which contributes to the increased flexural behavior at temperatures such as 400 °C [37]. After exposure to 1000 °C, the enlarged pores and voids can be observed, as shown in Figure 10b,f, which tends to weaken the matrix compactness and strength. However, a viscous sintering occurs at 1000 °C and generates ceramic-like phases, consequently significantly improving the residual flexural behavior and high temperature resistance. Furthermore, the corundum aggregates and their interfacial bond with the geopolymer matrix remain stable and well-bonded under both ambient and high-temperature conditions. This indicates that the design geopolymer exhibits excellent thermal compatibility between corundum aggregates and the matrix, as well as an inherent thermal stability of the aggregate framework compared with normal silica or limestone aggregate concrete [1,3,38]. The used wollastonite fibers could provide a strong bridge effect in the case of both ambient and high temperature exposures, as seen in Figure 9e and Figure 10d. The reinforced effect of wollastonite fibers at a microscale benefits microstructural crack resistance and residual flexural behavior [31].

**Figure 11:**
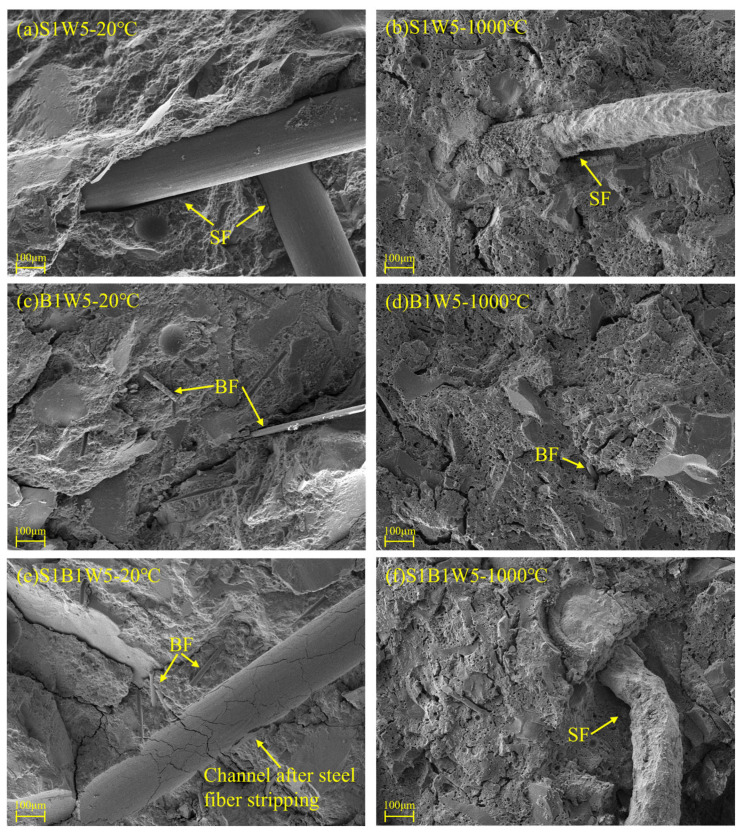
SEM morphologies of geopolymer with hybrid fibers: (**a**) S1W5-20°C, (**b**) S1W5-1000°C, (**c**) B1W5-20°C, (**d**) B1W5-1000°C, (**e**) S1B1W5-20°C and (**f**) S1B1W5-1000°C.

Figure 11 shows the SEM morphologies of geopolymer reinforced by binary and ternary hybrid fibers, both at ambient temperature (20 °C) and after exposure to 1000 °C. The hybrid fibers are dispersed in the geopolymer specimens and tightly bonded with the matrix at ambient conditions, as can be seen in Figure 11a,c,e. Obvious channels and scratches after steel fiber stripping can be observed, which indicates that the fibers provide a pullout force and efficiently inhibit the crack development, then enhance the flexural strength, deformation ability and toughness. Due to the excellent inherent thermal resistance, the basalt fibers can still remain in the geopolymer and give some bonding and bridging effects [7,17], as shown in Figure 11d. However, Figure 11b,f indicate that the steel fibers suffer from a severe oxidation reaction and deterioration of mechanical properties after 1000 °C exposures. Additionally, the steel fibers should undergo expansive volume and thermal incompatibility to the matrix at high temperature conditions [1], which certainly weaken the interfacial bonding performance. As a result, oxidized steel fibers become brittle and lose their reinforcing capacity, sometimes even contributing negatively to the residual flexural performance. These microstructural observations align well with the mechanical behavior presented in Figure 4.

## 4. Conclusions

This study investigates the flexural behavior of geopolymer composites reinforced with wollastonite, basalt and steel fibers at ambient and after high temperature exposure. The main conclusions can be drawn as follows:

(1) The designed geopolymer shows excellent residual flexural behavior using optimized metakaolin/fly ash blended precursors, a potassium silicate alkali activator and corundum aggregates. The flexural strength innovatively exhibits a continuously enhanced trend from 10 MPa at 20 °C to 25.9 MPa after 1000 °C exposure. This enhancement is mainly attributed to thermally induced further geopolymerization and ceramic-like crystalline phase formation due to viscous sintering.
(2) Introducing single 5% wollastonite fibers slightly increases the initial and residual flexural strength of geopolymer due to the thermal stability and microscale bridging effect, especially in the case of high temperature conditions. But it fails to change the residual flexural peak deflection, toughness and brittle failure.
(3) The utilization of binary wollastonite and basalt fibers in geopolymer generally results in similar and comparable initial and residual flexural load-displacement responses compared with the mixture with wollastonite fiber, while the residual flexural strength is obviously improved after 400–800 °C exposures compared with the reference geopolymer due to the inherent high temperature resistance of basalt fiber.
(4) The 5% wollastonite and 1% steel fiber-reinforced geopolymer presents remarkable improvements on flexural strength, deformation capacity and toughness before and after high temperature exposures. A higher steel fiber content in W5S2 could further reinforce the flexural strength and toughness of geopolymer exposed to 20–800 °C. The highest flexural strength and toughness occur after 400 °C exposure, namely at approximately 39.8 MPa and 43.7 N∙m, respectively. But too high a steel fiber content such as 2% even has a negative effect on flexural properties at 1000 °C exposure because of the severe oxidation deterioration and thermal incompatibility.
(5) The ternary hybrid fibers in geopolymers exhibit a positive synergistic effect on the flexural strength and toughness at 20–600 °C and maintain comparable properties at 800–1000 °C compared with the binary hybrid fibers in W5B1 and W5S1.

The designed hybrid fiber-reinforced geopolymers show excellent residual flexural behavior after high temperature exposure. Further research can be explored, including larger scale testing on structural components and fire resistance at a structural scale under steel rebar reinforcement.

## Figures and Tables

**Table 1:** Chemical composition of FA, MK and WF.

Oxide (%)	SiO_2_	Al_2_O_3_	CaO	MgO	Fe_2_O_3_	K_2_O	TiO_2_	Others
FA	60.91	31.09	3.29	0.38	0.3	1.29	0.92	1.02
MK	49.58	48.21	0.11	-	0.47	0.12	0.91	0.40
WF	48.67	0.24	49.74	0.69	0.37	0.04	-	0.25

**Table 2:** Mix design of the fiber-reinforced geopolymer (kg/m^3^).

Mix No.	FA	MK	CA	WG	Water	WF	BF	SF
F0	456.8	195.8	1762	252.4	99.9	0	0	0
W5	456.8	195.8	1762	252.4	99.9	32.6	0	0
W5B1	456.8	195.8	1762	252.4	99.9	32.6	27	0
W5S1	456.8	195.8	1762	252.4	99.9	32.6	0	78.5
W5S2	456.8	195.8	1762	252.4	99.9	32.6	0	157
W5B0.5S1	456.8	195.8	1762	252.4	99.9	32.6	13.5	78.5
W5B1S1	456.8	195.8	1762	252.4	99.9	32.6	27	78.5

FA: fly ash, MK: metakaolin, CA: corundum aggregate, WG: water glass, WF: wollastonite fiber, BF: basalt fiber, SF: steel fiber.

## Data Availability

The original contributions presented in this study are included in the article. Further inquiries can be directed to the corresponding authors.
